# A new species of *Docosia* Winnertz from Central Europe, with DNA barcoding based on four gene markers (Diptera, Mycetophilidae)

**DOI:** 10.3897/zookeys.549.6925

**Published:** 2016-01-05

**Authors:** Jan Ševčík, David Kaspřák, Björn Rulik

**Affiliations:** 1 University of Ostrava, Faculty of Science, Department of Biology and Ecology, Chittussiho 10, CZ-710 00 Ostrava, Czech Republic; 2 Silesian Museum, Nádražní okruh 31, CZ-746 01 Opava, Czech Republic; 3 Zoologisches Forschungsmuseum Alexander Koenig, Zentrum für Molekulare Biodiversitaetsforschung, Adenauerallee 160, D-53113 Bonn, Germany

**Keywords:** Bibionomorpha, Sciaroidea, fungus gnats, Palaearctic Region, taxonomy, DNA sequences, identification key

## Abstract

A new species of *Docosia* Winnertz, *Docosia dentata* **sp. n.**, is described and illustrated, based on a single male specimen collected in Muránska planina National Park in Central Slovakia. DNA sequences (COI, COII, CytB, and ITS2) are included and compared for 13 species of *Docosia*. There was found only little congruence between the molecular results and previous scarce data about interspecific relationships based on morphology. The COI and CytB gene markers showed the highest interspecific gene distances while ITS2 showed the lowest ones. An updated key to the 23 Central European species of *Docosia* is also presented.

## Introduction

The species in the genus *Docosia* Winnertz, 1863 are rather uniform, medium sized fungus gnats (Diptera: Mycetophilidae) with dark body and unmarked hyaline wings. Their identification is mainly based on the characters on the male and female terminalia (cf. Laštovka and Ševčík 2006). The genus was traditionally placed in the subfamily Leiinae (e.g. Søli 1997), though recent molecular studies challenge this and place *Docosia* among the Gnoristinae genera (Rindal et al. 2009, Ševčík et al. 2013).

Data on the biology of *Docosia* are scarce, with the exception of the common mycophagous species *Docosia gilvipes* (Walker, 1856), see the recent reviews by Chandler (2010), Jakovlev (2012) and Ševčík (2010a). One species (*Docosia fumosa* Edwards, 1925) has repeatedly been reared from bird’s nests (cf. Rulik and Kallweit 2006).

Although *Docosia* is a principally Holarctic genus, several species have also been reported from the Neotropical, Afrotropical and Oriental regions (Bechev 2000, Ševčík 2010b, Oliveira and Amorim 2011, Kurina and Ševčík 2012). A total of 32 described species are currently known from Europe (Kurina and Ševčík 2011). Central European species may be identified according to the key and illustrations of the male and female terminalia provided by Laštovka and Ševčík (2006). Their key includes 16 species from the Czech and Slovak Republics. Additional species were described and figured by Ševčík (2006) from Slovakia, by Ševčík and Laštovka (2008) and Kurina (2008) from the Alps, and by Kurina and Ševčík (2011) from Slovakia and Greece.

A further new species of *Docosia* from Slovakia, tentatively announced by Ševčík (2012), is described in this contribution, together with DNA sequence data provided for this and related species, and the opportunity is taken to update the key to the 23 Central European species of the genus. Morphological terminology follows that of Søli (1997).

## Material and methods

### Material from the Czech and Slovak Republics

The holotype of the new species was collected by the senior author by sweeping in Muránska planina National Park in central Slovakia. This national park belongs to the most valuable protected areas in Slovakia with regard to biodiversity, with many rare and thermophilous species, often reaching there the northernmost limit of their distribution (cf. Ševčík and Kurina 2011a, b). The habitats include mainly karst valleys and limestone rocky slopes, covered mostly by beech and spruce forests.

The material used for DNA extraction was collected with Malaise traps or sweep net at different localities of the Czech and Slovak Republics in the years 2012–2015. The voucher specimens are deposited in the collection of Jan Ševčík (JSEVC) or in the Silesian Museum, Opava, Czech Republic (SMOC).

All the specimens were collected in 70% ethanol. The holotype is stored in the glycerine medium in a plastic pinned microvial. Before placing in glycerol the specimen was incubated in proteinase K to extract DNA.

Genomic DNA was extracted using NucleoSpin Tissue Kit (Macherey-Nagel, Düren, Germany) following the included protocol. The specimens were cleaned with PBS (Phosphate Buffered Saline) and left in lysis buffer with proteinase K overnight at 56 °C. The primers used for the PCR amplifications were as follows: LCO1490 (5′-GGTCAACAAATCATAAAGATATTGG-3′) and HCO2198 (5′-TAAACTTCAGGGTGACCAAAAAATCA-3′) (Folmer et al. 1994) for COI; CYTB-F (5’-TATGTTTTATGAGGACAAATATC-3’) and CYTB-R (5’-AAATTCTATCTTATGTTTCAAAAC-3’) (Su et al. 2008) or mCYTB-R (5’-ATTACTCCCCCTAATTTATTAGGAAT-3’) (Ševčík lab) for cytochrome B; ITS2a (5’-TGTGAACTGCAGGACACAT-3’) and ITS2b (5’-TATGCTTAAATTCAGGGGGT-3’) (Beebe & Saul, 1995) for ITS2; and mCOII-F (5’-CAAGATAGAGCTTCTCCTCTTATAG-3’) and mCOII-R (5’-GGCATAAATCTATGATTAGCCCCAC-3’) (Ševčík lab) for COII. The amplification programme for the gene fragments was 94 °C for 3 min, followed by 35 cycles of 94 °C for 1 min, 49–50 °C for 1 min and 72 °C for 1:30 min and a final extension step at 72 °C for 7 min. The obtained PCR products were purified using Gel/PCR DNA Fragments Extraction Kit (Geneaid, New Taipei City, Taiwan) following manufacturer’s protocol and sequenced by Macrogen Europe (Netherlands).

### Material from Germany

German material discussed here was obtained within the German Barcode of Life Project (GBOL). Specimens used for DNA extraction were collected with Malaise traps at two different localities in Germany in the years 2013 and 2015. All the specimens were collected in pure 96% ethanol.

Genomic DNA was extracted from legs of the specimens using the BioSprint96 magnetic bead extractor by Qiagen (Hilden, Germany). Polymerase chain reaction (PCR) was carried out in total reaction mixes of 20 μl, including 2 μl of undiluted DNA template, 0,8 μl of each primer (10 pmol/μl), 2 μl of ‘Q-Solution’ and 10 μl of ‘Multiplex PCR Master Mix’, containing hot start Taq DNA polymerase and buffers. The latter components are available in the Multiplex PCR kit from Qiagen (Hilden, Germany). PCR reactions were run individually and not multiplexed.

Thermal cycling was performed on GeneAmp PCR System 2700 (Applied Biosystems, Foster City, CA, USA) as follows: hot start Taq activation: 15 min at 95 °C; first cycle set (15 repeats): 35-s denaturation at 94 °C, 90-s annealing at 55 °C (−1 °C/cycle) and 90-s extension at 72 °C. Second cycle set (25 repeats): 35-s denaturation at 94 °C, 90-s annealing at 40 °C and 90-s extension at 72 °C; final elongation 10 min at 72 °C using the primers LCO1490-JJ: 5´-CHACWAAYCATAAAGATATYGG- 3´ with HCO2198-JJ: 5´-AWACTTCVGGRTGVCCAAARAATCA- 3´ respectively (Astrin and Stüben 2008).

Sequencing of the unpurified PCR products in both directions was conducted at Beijing Genomics Institute (Hongkong, CN) by using the amplification primers. Barcode sequence analysis was done using the Geneious® software version 7.1.7 (http://www.geneious.com).

Vouchers were deposited in the collection of the Zoologisches Forschungsmuseum Alexander Koenig, Bonn, Germany.

### Sequence alignment and analyses

The sequences were aligned using MAFFT version 7 (Katoh and Standley 2013) on the MAFFT server (http://mafft.cbrc.jp/alignment/server/) with default settings and then manually edited. The protein-coding gene COI, COII and CytB sequences were checked based on amino-acid translations and yielded indel-free nucleotide alignments. All unreliably aligned regions of ITS2 fragment were removed in GBLOCKS 0.91b program (Castresana 2000) on the Gblocks server (http://molevol.cmima.csic.es/castresana/Gblocks_server.html). We created four alignments, one for each gene, and one concatenated alignment for all gene fragments with 15 taxa, including additional sequences from GenBank (KT316839, KC435639, KC435683 and KC435708). The final molecular dataset consists of 2039 characters: COI–658, COII–546, CytB–433, ITS2–402 bp. The datasets were analysed using maximum likelihood analyses conducted on the CIPRES computer cluster using RAxML-HPC BlackBox 7.6.3 (Stamatakis 2006) employing automatic bootstrapping on the partitioned dataset. All the sequences were deposited into GenBank and BOLD (http://dx.doi.org/10.5883/DS-DOCODENT), where detailed metadata is available (see Table 1). As outgroup taxa we selected two representatives of the subfamily Gnoristinae, in concordance with previous molecular studies (Rindal et al. 2009, Ševčík et al. 2013).

Genetic distances were calculated in MEGA6 (Tamura et al. 2013) using Kimura 2-parameter model (K2P) with pairwise deletion for the treatment and they are demonstrated in Table 2 and Fig. 5.

**Table 1.** List of species, sampling locality and year, and accession numbers.

Species	Voucher code	Sampling locality and year	GenBank accession numbers				BOLD Process ID
			COI	COII	CYTB	ITS2	
*Boletina nasuta* (Haliday, 1839)	JSGS18	Slovakia, 2013	KT923571	KT923585	KT923598	KT923614	JSDO011-15
*Gnoriste bilineata* Zetterstedt, 1852	JSGS4	Czech Republic, 2009	KT316839	KT923584	KT923597	KT923613	JSDO014-15
*Docosia dentata* sp. n.	JSDO1	Slovakia, 2012	KT923562	KT923575	KT923600	KT923604	JSDO001-15
*Docosia diutina* Plassmann, 1996	ZFMK-TIS-2516913	Germany, 2013	KU146854	KU146860	KU146858	KU146856	SRDOC001-15
*Docosia flavicoxa* Strobl, 1900	JSDO9a	Slovakia, 2012	KT923570	KT923583	KT923596	KT923612	JSDO009-15
*Docosia fumosa* Edwards, 1925	ZFMK-TIS-2556735	Germany, 2015	KU146855	KU146861	KU146859	KU146857	SRDOC002-15
*Docosia fuscipes* (Roser, 1840)	JSDO2	Slovakia, 2015	KT923563	KT923576	KT923590	KT923605	JSDO002-15
*Docosia gilvipes* (Walker, 1856)	JSGS29	Slovakia, 2013	KT923572	KT923586	KT923599	KT923615	JSDO012-15
*Docosia landrocki* Laštovka & Ševčík, 2006	JSDO7	Slovakia, 2014	KT923568	KT923581	KT923594	KT923610	JSDO007-15
*Docosia lastovkai* Chandler, 1994	JSDO4	Slovakia, 2013	KT923565	KT923578	KT923591	KT923607	JSDO004-15
*Docosia montana* Laštovka & Ševčík, 2006	JSDO5	Slovakia, 2013	KT923566	KT923579	KT923592	KT923608	JSDO005-15
*Docosia moravica* Landrock, 1916	JSDO6	Slovakia, 2013	KT923567	KT923580	KT923593	KT923609	JSDO006-15
*Docosia muranica* Kurina & Ševčík, 2011	JSM10	Slovakia, 2013	KC435639	KT923587	KC435683	KC435708	JSDO013-15
*Docosia sciarina* (Meigen, 1830)	JSDO8	Czech Republic, 2014	KT923569	KT923582	KT923595	KT923611	JSDO008-15
*Docosia setosa* Landrock, 1916	JSDO3	Slovakia, 2015	KT923564	KT923577	KT923601	KT923606	JSDO003-15

## Species description

### Docosia dentata sp. n.

Taxon classificationAnimaliaDipteraMycetophilidae

http://zoobank.org/E75577BA-83A0-4596-BC12-61F22A505225

Figures 1–3

#### Type material.

Holotype male, in a pinned microvial with glycerol. SLOVAKIA, Muránska planina National Park, Muráň env., Šiance National Nature Reserve, sweeping along forest edge, N48°46'12", E20°04'20", 1005 m a.s.l., 25. May 2012 (J. Ševčík leg.) [SMOC].

#### Description.

Male (n = 1). Length of wing 4.2 mm.

Head blackish brown with numerous pale setae. Three ocelli, with lateral ones almost touching compound eyes, separated from the eye margins by their own diameter. Clypeus blackish, with setae pale. Mouthparts light brownish. Palpus brownish yellow, basally and apically darker. Scape, pedicel and all flagellomeres dark brown. Flagellomeres cylindrical, flagellomeres 1 to 7 about twice as long as broad, apical flagellomeres (8 to 14) slightly conical, three times as long as broad.

All parts of thorax blackish brown. All bristles and setae yellowish white. Scutellum with several marginal and submarginal pale bristles and with numerous setae. Antepronotum and proepisternum with pale bristles and short dark setae. Upper part of antepronotum with a strong pale bristle reaching to the ocellus. Laterotergite and other pleural parts bare. Haltere pale yellow.

Legs. All coxae entirely yellow. Femora yellow with hind femur brownish only around its tip. All trochanters blackish brown. Tibiae and tarsi yellow, tarsal segments seemingly brownish because of dense setulae. Fore tibia apicomedially with a semicircular tibial organ (anteroapical depressed area), without strong setae, only densely covered with fine setulae. Mid tibia with 5 anterior, 4 dorsal, 3 anteroventral and 5–6 posterior setae. Hind tibia with 16 anterior, 12 dorsal, 5 anteroventral and 6 posterior setae.

Wings hyaline, unmarked. Radial veins and r-m brown, other veins paler while m-stem and the base of M_1_ are faint, almost not traceable. Sc, Rs and basal third of cu-stem asetose, the other veins setose. Costa reaches to 0.45 of the distance between R_5_ and M_1_. Sc ends in R at the level of beginning of m-stem. Posterior fork begins before anterior fork, approximately at the level of the middle of r-m.

**Figures 1–3.**
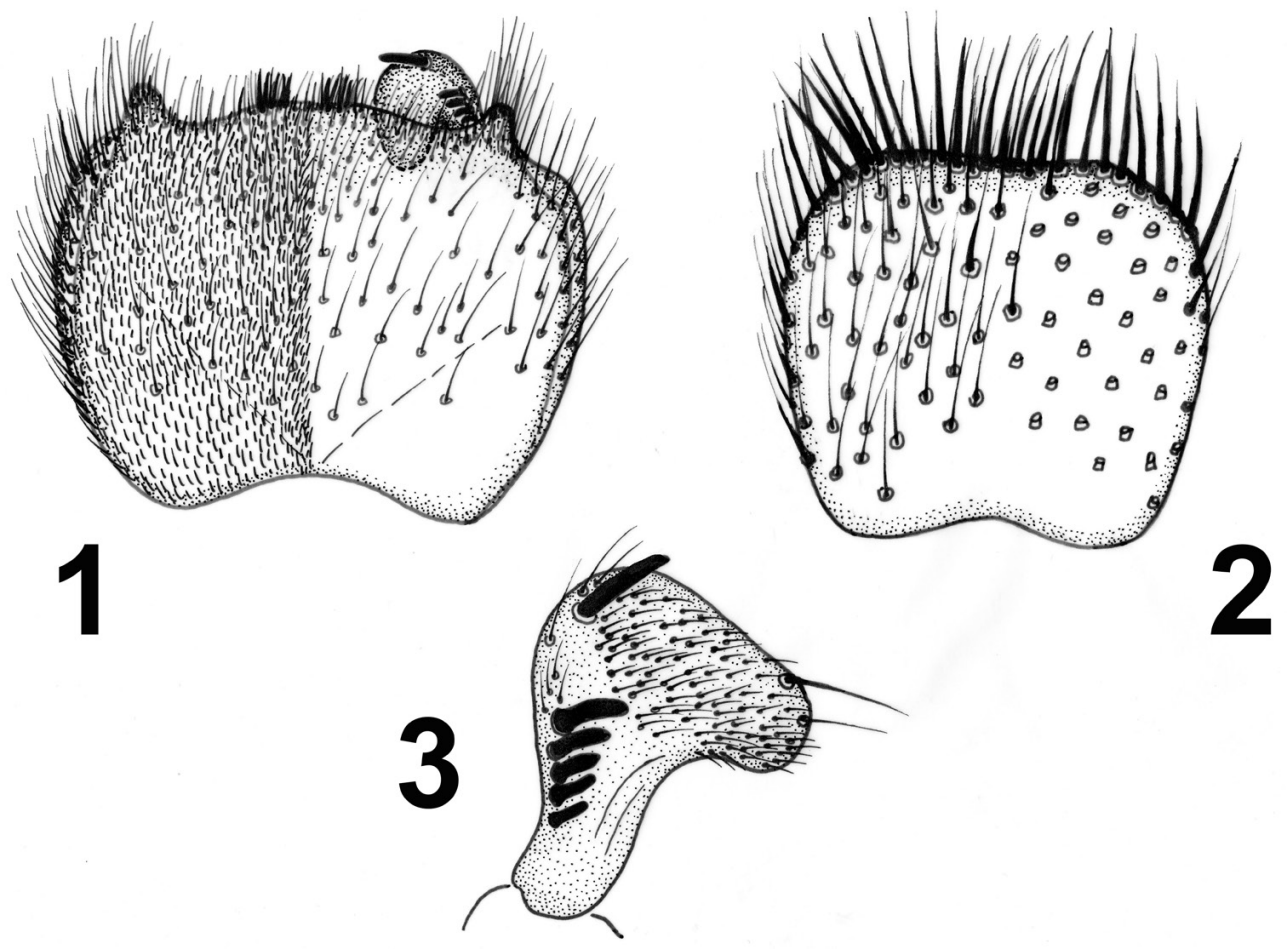
*Docosia dentata* sp. n. (holotype), male terminalia; **1** ventral view **2** tergite 9 in dorsal view **3** right gonostylus in dorsal view. Scale bar = 0.1 mm.

Abdomen all dark brown. Terminalia (Figs 1–3) dark brown except lighter gonostyli. Tergite 9 in the shape of a rounded square, about as long as broad (Fig. 2). Posteroventral margin of gonocoxites with lateral projections and with two patches of short setae medially (Fig. 1). Gonostylus subtriangular with a row of black megasetae along its ventral margin (Fig. 3).

**Female.** Unknown.

#### Biology.

Unknown.

#### Etymology.

The species name refers to the structure of gonostylus.

### Molecular data

The genetic distances among the 13 species of *Docosia* and two outgroup species for particular gene markers are presented in Tab. 2. The interspecific differences among *Docosia* species ranged from 0.3% (in ITS2 between *Docosia dentata* and *Docosia muranica*) to 26.0% (in CytB between *Docosia fumosa* and *Docosia lastovkai*), with the mean interspecific distances for particular genes: 12.8% (COI), 10.3% (COII), 14.9% (CytB) and 4.5% (ITS2). The genetic distances for the nuclear ITS2 marker were remarkably lower than for the mitochondrial markers. Fig. 5 shows that the distribution of K2P distances for cytB is rather broad (6.6–28%), while the distributions for COI, COII and ITS2 are relatively narrow.

**Table 2.** Kimura 2-parametr genetic distances for COI, COII, CytB and ITS2. The lowest and highest values among *Docosia* species are highlighted in bold.

	**COI**	1	2	3	4	5	6	7	8	9	10	11	12	13	14
1	*Docosia dentata* sp. n.														
2	*Docosia diutina*	8,6%													
3	*Docosia flavicoxa*	9,8%	12,4%												
4	*Docosia fumosa*	13,3%	13,1%	13,5%											
5	*Docosia fuscipes*	8,2%	9,4%	11,1%	12,8%										
6	*Docosia gilvipes*	13,9%	14,6%	14,1%	12,8%	13,5%									
7	*Docosia landrocki*	9,3%	9,9%	12,3%	13,3%	10,6%	15,9%								
8	*Docosia lastovkai*	12,6%	13,5%	15,2%	13,9%	11,9%	14,8%	14,0%							
9	*Docosia montana*	7,5%	10,6%	11,4%	15,3%	10,4%	14,8%	11,7%	14,4%						
10	*Docosia moravica*	13,2%	15,1%	15,4%	19,1%	14,7%	**19,6**%	15,1%	17,0%	16,4%					
11	*Docosia muranica*	10,2%	13,0%	14,6%	16,6%	11,3%	16,2%	13,3%	15,5%	12,0%	17,8%				
12	*Docosia sciarina*	9,4%	11,5%	11,8%	14,6%	11,2%	14,3%	12,0%	13,1%	9,0%	15,7%	14,4%			
13	*Docosia setosa*	**6,0**%	10,0%	10,3%	14,1%	10,3%	14,7%	10,6%	13,9%	7,1%	14,7%	12,3%	9,0%		
14	*Boletina nasuta*	15,9%	17,8%	16,2%	18,6%	16,3%	16,1%	17,4%	16,4%	18,0%	20,1%	16,2%	15,7%	16,1%	
15	*Gnoriste bilineata*	16,1%	17,9%	18,4%	15,4%	17,0%	15,6%	18,2%	17,5%	18,6%	19,8%	20,3%	17,5%	17,2%	14,9%
															
	**COII**	1	2	3	4	5	6	7	8	9	10	11	12	13	14
1	*Docosia dentata* sp. n.														
2	*Docosia diutina*	6,6%													
3	*Docosia flavicoxa*	7,5%	8,2%												
4	*Docosia fumosa*	14,4%	17,2%	14,9%											
5	*Docosia fuscipes*	7,5%	8,9%	11,0%	17,4%										
6	*Docosia gilvipes*	7,2%	10,2%	11,9%	16,8%	8,6%									
7	*Docosia landrocki*	6,8%	5,6%	7,8%	15,2%	8,4%	10,7%								
8	*Docosia lastovkai*	11,7%	12,9%	14,2%	**19,3**%	11,7%	9,7%	14,8%							
9	*Docosia montana*	6,2%	7,3%	9,4%	15,8%	9,3%	10,3%	8,4%	13,5%						
10	*Docosia moravica*	6,2%	8,8%	7,3%	16,6%	9,1%	9,3%	9,1%	12,2%	7,3%					
11	*Docosia muranica*	8,6%	11,0%	12,9%	16,0%	9,1%	**4,3**%	11,5%	12,2%	11,1%	11,0%				
12	*Docosia sciarina*	6,7%	8,7%	8,4%	17,4%	10,1%	8,9%	8,9%	13,0%	6,9%	7,3%	11,6%			
13	*Docosia setosa*	5,1%	6,4%	7,7%	15,2%	8,2%	10,0%	6,4%	13,5%	5,6%	6,6%	11,0%	7,4%		
14	*Boletina nasuta*	23,1%	22,8%	20,9%	21,9%	21,2%	22,4%	22,4%	23,1%	21,2%	22,2%	20,0%	22,6%	21,0%	
15	*Gnoriste bilineata*	17,7%	18,5%	18,2%	18,2%	17,7%	19,7%	18,8%	25,4%	18,8%	18,0%	17,4%	18,6%	17,9%	19,1%
															
	**cytB**	1	2	3	4	5	6	7	8	9	10	11	12	13	14
1	*Docosia dentata* sp. n.														
2	*Docosia diutina*	8,3%													
3	*Docosia flavicoxa*	11,0%	14,5%												
4	*Docosia fumosa*	19,3%	22,0%	21,0%											
5	*Docosia fuscipes*	10,9%	12,5%	14,9%	24,1%										
6	*Docosia gilvipes*	19,6%	20,6%	19,9%	19,7%	21,9%									
7	*Docosia landrocki*	10,1%	9,7%	14,3%	23,6%	12,2%	19,9%								
8	*Docosia lastovkai*	15,5%	16,4%	15,8%	**26,0**%	18,7%	21,0%	15,4%							
9	*Docosia montana*	9,4%	12,8%	17,1%	22,9%	13,1%	22,3%	13,5%	21,9%						
10	*Docosia moravica*	10,0%	11,6%	12,0%	17,8%	14,0%	19,5%	12,0%	16,1%	14,5%					
11	*Docosia muranica*	7,8%	12,6%	13,6%	21,6%	10,4%	19,4%	12,9%	16,2%	13,1%	13,9%				
12	*Docosia sciarina*	7,8%	11,0%	10,8%	21,7%	11,4%	19,9%	10,1%	16,1%	11,6%	10,5%	10,7%			
13	*Docosia setosa*	**6,6**%	10,3%	10,0%	21,5%	11,0%	16,7%	9,4%	15,7%	10,8%	10,3%	10,2%	7,4%		
14	*Boletina nasuta*	17,4%	18,8%	19,0%	21,9%	22,2%	18,9%	22,3%	20,8%	25,0%	17,9%	23,9%	18,5%	19,9%	
15	*Gnoriste bilineata*	16,0%	18,8%	19,0%	18,9%	20,6%	20,4%	20,3%	21,1%	21,2%	16,0%	20,2%	18,9%	18,4%	17,1%
	**ITS2**	1	2	3	4	5	6	7	8	9	10	11	12	13	14
1	*Docosia dentata* sp. n.														
2	*Docosia diutina*	1,8%													
3	*Docosia flavicoxa*	2,6%	3,4%												
4	*Docosia fumosa*	9,6%	9,2%	10,4%											
5	*Docosia fuscipes*	0,8%	2,3%	3,5%	8,7%										
6	*Docosia gilvipes*	7,2%	6,9%	9,1%	11,3%	7,2%									
7	*Docosia landrocki*	2,0%	1,3%	3,7%	9,2%	2,9%	6,0%								
8	*Docosia lastovkai*	4,3%	5,2%	5,8%	**12,7**%	4,7%	7,9%	5,2%							
9	*Docosia montana*	2,0%	3,4%	3,2%	9,6%	2,1%	8,7%	3,7%	6,4%						
10	*Docosia moravica*	1,3%	3,2%	1,8%	9,2%	1,9%	8,3%	3,5%	4,4%	2,6%					
11	*Docosia muranica*	**0,3**%	2,1%	2,9%	9,6%	0,5%	6,9%	2,3%	4,1%	2,3%	1,3%				
12	*Docosia sciarina*	1,3%	2,1%	3,4%	9,4%	2,1%	7,9%	2,3%	5,8%	2,1%	2,6%	1,5%			
13	*Docosia setosa*	1,3%	2,1%	2,3%	9,0%	2,1%	8,2%	2,3%	5,8%	1,8%	2,4%	1,5%	1,0%		
14	*Boletina nasuta*	23,7%	23,3%	23,6%	22,0%	23,4%	21,2%	23,3%	25,1%	23,2%	23,1%	23,3%	21,7%	21,7%	
15	*Gnoriste bilineata*	20,5%	20,5%	20,3%	19,3%	20,2%	20,1%	20,5%	21,1%	19,6%	19,7%	20,1%	19,0%	18,5%	12,8%

**Figure 4.**
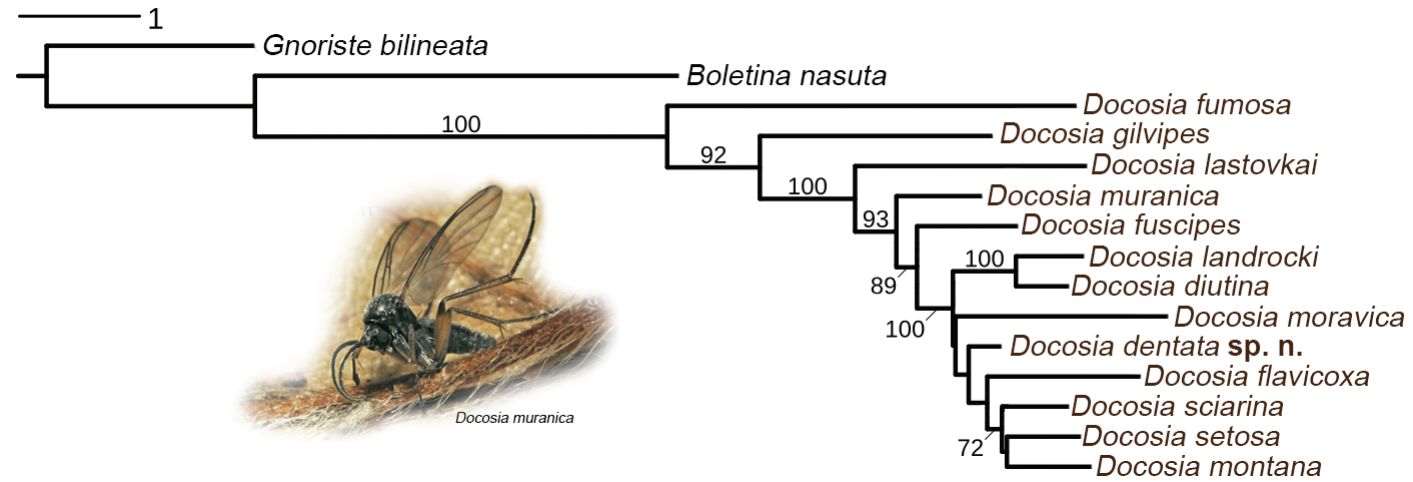
Maximum likelihood hypothesis for relationships among selected species of *Docosia* Winnertz based on DNA sequence data (COI, COII, CytB and ITS2), 2039 characters. Above node number = bootstrap support for ML.

The phylogenetic tree for the concatenated dataset is presented in Fig. 4. The genus *Docosia* was found to be monophyletic with maximum bootstrap support value (BV = 100). Surprisingly, *Docosia fumosa* branched basally as a sister group to all the other *Docosia* species included in this analysis, followed by *Docosia gilvipes*. All the other *Docosia* species grouped together as a monophyletic group with maximum support (BV = 100). Within this group, only *Docosia diutina* and *Docosia landrocki* were found to be closely related (BV = 100) while the other relationships between any two species are less supported. Also the monophyly of the group comprising eight terminal species (see Fig. 4) is highly supported (BV = 100). A sister taxon to this group is *Docosia fuscipes*, while *Docosia muranica* is sister taxon to the entire latter group. Finally, *Docosia lastovkai* is sister taxon to the all *Docosia* species in this dataset, except *Docosia fumosa* and *Docosia gilvipes*.

**Figure 5.**
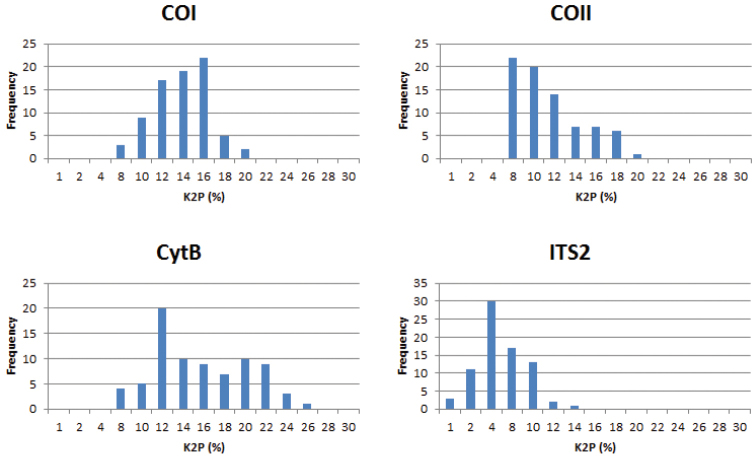
Frequency distributions of genetic distances (K2P) for particular gene markers.

## Discussion

### Affiliation of the new species

According to the key by Laštovka and Ševčík (2006), the new species runs to couplet 10 (*Docosia montana* Laštovka & Ševčík, 2006), because of bare laterotergites, yellow coxae and basal flagellomeres relatively long. The structure of the male terminalia is, however, quite different from this species and also from two other species described later (*Docosia chandleri* Ševčík & Laštovka, 2008 and *Docosia matilei* Ševčík & Laštovka, 2008), which would run to couplet 10 too. It is thus difficult to find the closest relative of *Docosia dentata* according to morphological characters.

Considering the genetic distance, the closest relative of *Docosia dentata* is *Docosia setosa*, based on COI (6.0 %), COII (5.1%) and CytB (6.6%) gene markers. In the phylogenetic tree based on all the four gene markers (Fig. 4), *Docosia dentata* forms a sister branch to a group of the following four species: *Docosia flavicoxa*, *Docosia sciarina*, *Docosia montana*, and *Docosia setosa*.

### Comparison of the utility of COI, COII, CytB and ITS2 for barcoding of *Docosia*

The comparison of the four variable gene regions revealed that the genetic distances between *Docosia* species are quite high for CytB and COI, with the average values being 14.9% and 12.8%, respectively. From this point of view, CytB performs as the best barcoding marker for *Docosia* species, followed by the traditional animal barcoding region (COI), COII and ITS2. The ITS2 sequences show remarkably high similarity in the genus *Docosia* and do not appear as a suitable barcode marker in this case. The high uniformity of ITS2 has been reported in several studies (e.g. Lv et al. 2014 or Navajas et al. 1988). At least within Mycetophilidae, the ITS2 marker may thus possibly be more suitable for intergeneric comparisons and higher phylogeny, similarly as the neighbouring ribosomal 28S region. In mycetophilids, ITS2 has already been successfully used for inferring phylogeny at generic or subgeneric level by Ševčík et al. (2013, 2014). This issue definitely deserves further study.

There are many studies comparing the utility of various gene markers for DNA barcoding and the identification of species (e.g. Bourke et al. 2013, Lv et al. 2014, Schwarzfeld and Sperling 2014). Concerning fungus gnats, comparisons between COI and ITS2 were recently provided by Jürgenstein et al. (2015) and Kurina et al. (2015). They both came to the conclusion that COI performed better.

The CytB region has mostly been used in the studies devoted to vertebrates but it was recently used also for barcoding of Calliphoridae (GilArriortua et al. 2013), tse-tse flies (Orji et al. 2015) or aphids (Chen et al. 2012).

### Incongruence between molecular and morphological data

There has been no previous phylogenetical hypothesis for *Docosia* species published to date but if we take into account the characters used in most available keys, there is only little congruence between those morphological characters and the molecular results presented here. For example, *Docosia fumosa* does not appear as particularly separated from the other species by morphological characters. It belongs to the group of species with pubescent lateroterites, together with *Docosia flavicoxa*, *Docosia gilvipes*, *Docosia moravica*, *Docosia sciarina*, and several other species, not represented in our molecular dataset. These latter species do not constitute a monophyletic group in the molecular tree (Fig. 4) nor do the species with bare laterotergites. This means that this widely used character (pubescence of laterotergites) has most probably no or little value in reconstructing relationships within the genus.

The second widely used character in the keys is the coloration of legs, mainly the coxae, which also does not help much in defining any group congruent with the clades in the molecular tree presented here.

Further, it is difficult to find clear morphological synapomorphies for the highly supported clades in the tree (Fig. 4), e.g. for the sister relationship of *Docosia diutina* + *Docosia landrocki* (except for the overall resemblance of their male terminalia).

Interestingly, in two of the four trees based on one gene region only (in COI and ITS2, data not shown), *Docosia gilvipes* (not *Docosia fumosa*) branched basally as a sister group to the rest of *Docosia*, a result which would be expected on the basis of morphological and ecological data (*Docosia gilvipes* is the only species in the dataset with Sc setose and ending free, different structure of male terminalia, endomycophagous larvae etc.).

Nevertheless, the number of species included in this analysis is rather limited to reach any final conclusion. A more comprehensive phylogeny of the genus, as well as of other genera of fungus gnats, is thus needed.

### Key to Central European species of Docosia

**Table:** 

1	Laterotergite pubescent	**2**
–	Laterotergite bare	**9**
2 (1)	Sc setose and ending free	**3**
–	Sc bare and ending in R	**4**
3 (2)	Tergite 9 subrectangular, gonostylus with a patch of fine subapical setae	***Docosia gilvipes* (Walker, 1856)**
–	Tergite 9 distinctly broadened posteriorly, gonostylus without a patch of subapical setae (Kurina 2008: figs 10–15)	***Docosia pseudogilvipes* Kurina, 2008**
4 (2)	Hind femur completely black or dark brown	**5**
–	Hind femur at least partly yellow	**6**
5 (4)	All coxae and palpi black	***Docosia carbonaria* Edwards, 1941**
–	Coxae yellowish brown, wings slightly smoked	***Docosia fumosa* Edwards, 1925**
6 (4)	Coxae largely dark (at least basal ½), laterotergal setae short	***Docosia sciarina* (Meigen, 1830)**
–	Coxae at most parts yellow, laterotergal setae long as usua	**7**
7 (6)	Mid tibia dorsally with longitudinal black band	***Docosia tibialis* Laštovka & Ševčík, 2006**
–	Mid tibia dorsally yellowish	**8**
8 (7)	Hind coxa basally brown, apical 1/5 and proximoventral part of hind femur darkened	***Docosia moravica* Landrock, 1916**
–	Hind coxa at most with basal margin darkened, hind femur dark only at tip, its ventral surface yellow	***Docosia flavicoxa* Strobl, 1910**
9 (1)	All coxae dark brown, hind femur largely dark	**10**
–	Coxae with at least apical half pale	**11**
10 (9)	Haltere yellowish, male gonocoxites broadly rounded posteriorly (Laštovka and Ševčík 2006: Fig. 7)	***Docosia fuscipes* (von Roser, 1840)**
–	Haltere with a dark knob, male gonocoxites with a deep median incision (Chandler and Blasco-Zumeta 2001: figs 42–47)	***Docosia morionella* Mik, 1884**
11 (9)	All coxae yellow, hind coxa dark at most on basal 1/6	**12**
–	Hind coxa blackish brown at least on the basal third	**17**
12 (11)	Antenna with long basal segments, flagellomere 1 to 4 about twice as long as wide, lateral ocelli distant from eyes	**13**
–	Antennal segments shorter, flagellomere 1 to 4 about 1.5 as long as wide, ocelli touching eyes	**16**
13 (12)	Pedicel yellow	***Docosia chandleri* Ševčík & Laštovka, 2008**
–	Pedicel dark brown	**14**
14 (13)	Posteroventral margin of gonocoxites almost straight, with posterolateral projections, gonostylus short, subtriangular (Figs 1, 3)	***Docosia dentata* sp. n.**
–	Posteroventral margin of gonocoxites medially with a distinct rounded projection, gonostylus elongated	**15**
15 (14)	Gonostylus with several unusually thick black megasetae along its ventral margin (Ševčík and Laštovka 2008: fig. 2)	***Docosia matilei* Ševčík & Laštovka, 2008**
–	Gonostylus with black megasetae only apically (Laštovka and Ševčík 2006: fig. 10)	***Docosia montana* Laštovka & Ševčík, 2006**
16 (12)	Costa extends to ½ distance from R_5_ to M_1_ or slightly more, apical 1/5 of hind femur dark	***Docosia lastovkai* Chandler, 1994**
–	Costa extends only to 2/5 distance from R_5_ to M_1_, only tip of hind femur dark	***Docosia landrocki* Laštovka & Ševčík, 2006**
17 (11)	Hind coxa dark only on basal third, haltere entirely yellowish	**18**
–	Basal half or slightly more of hind coxa blackish brown	**19**
18 (17)	Lateral ocelli distant from eyes (about a diameter of ocellus), mid coxa dark on basal third, pedicel dark, male tergite 9 subtrapezoidal (Laštovka and Ševčík 2006: fig. 15)	***Docosia setosa* Landrock, 1916**
–	Lateral ocelli touching eyes, mid coxa dark on basal fourth or less, pedicel usually pale, male tergite 9 subcircular (Laštovka and Ševčík 2006: fig. 4)	***Docosia expectata* Laštovka & Ševčík, 2006**
19 (17)	Costa extends about 2/5 from R_5_ to M_1_, palpi yellow, haltere darkened	***Docosia nigra* Landrock, 1928**
–	Costa extends only about 2/7 from R_5_ to M_1_, palpi darkened, haltere yellow	**20**
20 (19)	Male terminalia with tergite 9 short, as long as broad, trapezoid, posterior margin of gonocoxites without distinct medioventral process (Kurina and Ševčík 2011: fig. 2)	***Docosia muranica* Kurina & Ševčík, 2011**
–	Male terminalia with tergite 9 long, about twice as long as broad, posterior margin of gonocoxites with distinct medioventral process, hind tibia with a dark patch of modified setae	**20**
21 (20)	Male terminalia with lateral lobes of gonocoxites in ventral view distinctly longer than medioventral process of gonocoxites (Laštovka and Ševčík 2006: fig. 3)	***Docosia diutina* Plassmann, 1996**
–	Male terminalia with lateral lobes of gonocoxites shorter, at most as long as medioventral process of gonocoxites	**21**
22 (21)	Tergite 9 pear-shaped, distinctly broader in posterior half (Laštovka and Ševčík 2006: fig. 13)	***Docosia pannonica* Laštovka & Ševčík, 2006**
–	Tergite 9 subrectangular, not distinctly broader in posterior half (Ševčík 2006: fig. 3)	***Docosia rohaceki* Ševčík, 2006**

## Supplementary Material

XML Treatment for Docosia dentata
